# Implementing the 2024 revision of the McDonald diagnostic criteria for multiple sclerosis in Germany: Practical considerations and consensus recommendations

**DOI:** 10.1177/17562864261481602

**Published:** 2026-08-25

**Authors:** Mike P. Wattjes, Antonios Bayas, Stefan Bittner, Birte Elias-Hamp, Ralf Linker, Daniela Rau, Thomas Skripuletz, Ralf Gold

**Affiliations:** 1 Department of Neuroradiology, Charité - Universitätsmedizin Berlin, Corporate Member of Freie Universität Berlin, Humboldt-Universität zu Berlin, Berlin, Germany; 2 Department of Neurology and Clinical Neurophysiology, Faculty of Medicine, University of Augsburg, Augsburg, Germany; 3 Department of Neurology, Research Center for Immunotherapy (FZI) and Focus Program Translational Neuroscience (FTN), Rhine-Main Neuroscience Network (rmn2), University Medical Center of the Johannes Gutenberg University Mainz, Mainz, Germany; 4 Neurological Practice, Hamburg, Germany; 5 Department of Neurology, University Clinic at the Regensburg District Hospital, Regensburg, Germany; 6 Neurological Specialist Group Practice, Ulm, Germany; 7 Department of Neurology, 9177 Hannover Medical School, Hannover, Germany; 8 Department of Neurology, St. Josef Hospital, Ruhr University Bochum, Bochum, Germany

**Keywords:** multiple sclerosis, magnetic resonance imaging, central vein sign, paramagnetic rim lesions, optic nerve, McDonald criteria, cerebrospinal fluid, kappa free light chains

## Abstract

Early and reliable diagnosis of multiple sclerosis (MS) is critical for initiating an appropriate disease-modifying therapy (DMT). The 2024 revisions of the McDonald diagnostic criteria incorporate novel fluid and imaging biomarkers aiming to facilitate an accurate and timely diagnosis. The aim of this point-of-view paper is to discuss the implementation of the 2024 McDonald criteria in the clinical routine setting in context of the German Health care system and provide consensus recommendations by an interdisciplinary expert panel. A multidisciplinary panel of nine experts in the field of diagnosis and treatment of MS convened to discuss the potential advances and challenges of the clinical implementation of the 2024 McDonald criteria and develop consensus recommendations. The panel identified implementation challenges regarding limited specialized resources for the suggested additional imaging markers such as the detection of optic nerve (ON) lesions, paramagnetic rim lesions (PRLs) and the demonstration of lesions with a central vein sign (CVS) as well as the implementation of kappa free light chains (kFLC) in Germany. We advocate a stratified diagnostic approach: cerebrospinal fluid (CSF) analysis remains essential. The consideration of ON lesions, PRLs and lesions with a CVS should be reserved for diagnostic situations in which the diagnosis cannot be established with conventional MRI protocols or other methods for examination of the optic nerve. To minimize misdiagnosis and to address the complexity of treating radiologically isolated syndrome, there is a necessity of continuous professional training and nuanced, patient-centered therapeutic strategies, especially for individuals with radiologically isolated syndrome but also for atypical presentations. In conclusion, the 2024 revisions of the McDonald criteria aim to facilitate an accurate and early diagnosis of MS potentially enabling earlier MS therapy initiation. However, the routine implementation, particularly regarding the new imaging markers, requires an expansion of resources and expertise limiting the general applicability in the German healthcare system.

## Plain Language Summary

The new 2024 revisions of the McDonald criteria aim to faciliate an earlier and more accurate diagnosis of multiple sclerosis by implementing new magentic resonance imaging (MRI) and laboratory markers. However, the use of these MRI markers in the clinical routine setting require dedicated expertise and resources and have not been validated so far. Therefore, it remains unclear how and to which extend these criteria can be implemented in everyday clinical practice world wide, particularly in health care systems of countries with limited resources including Germany. A panel of nine MS experts, including neurologists and neuroradiologists, met to discuss the advantages and challenges of applying the 2024 McDonald criteria in the clinical routine care. Based on these discussions, the panel developed consensus recommendations for clinical practice in Germany. The experts identified several challenges related to the implementation of the new diagnostic markers. Additional MRI markers, such as optic nerve lesions, paramagnetic rim lesions, and lesions with a central vein sign, require an extension of the acquisition protocols and image analysis as well as an dedicated expertise which is not widely available. The use of kappa free light chains in laboratory testing is also not yet fully established in Germany. The panel recommended that cerebrospinal fluid analysis should remain a core part of MS diagnosis, while the newer imaging markers should mainly be used in inconclusive cases where standard MRI measures are not sufficient to establish the diagnosis of MS. The experts also stressed the importance of ongoing professional training and individualized treatment decisions, especially for patients with unusual findings or radiologically isolated syndromes. The updated McDonald criteria aim to facilitate an earlier MS diagnosis leading to an earlier treatment. However, the use of the proposed additional imaging and laboratory methods in routine care will require more dedicated resources.

## Introduction

Multiple sclerosis (MS) is the most common chronic inflammatory demyelinating disease in young adults and leads to long-term disability.^1^ An early and accurate diagnosis is essential, as disease-modifying therapies (DMT) administered at an early stage of MS have the potential to prevent relapses, downregulate highly aggressive immune cells and significantly slow the progression of disability.^2^ Since the introduction of the initial McDonald diagnostic criteria for relapsing-remitting MS in 2001, these criteria have continuously evolved to enhance sensitivity while remaining specificity facilitating an earlier and accurate diagnoses.^3,4^ The 2024 revision of the McDonald criteria incorporates new imaging and cerebrospinal fluid (CSF) biomarker aiming to allow an even earlier but still accurate diagnosis.^5^ However, these revisions also increase complexity by introducing previously not routinely used magnetic resonance imaging (MRI) and CSF biomarkers, as well as expand criteria to include asymptomatic patients and provide additional recommendations and considerations for patients beyond the age of 50 years as well as for pediatric-onset of MS.^4^

### Key updates in the 2024 McDonald criteria for the diagnosis of MS

**Figure 1.**
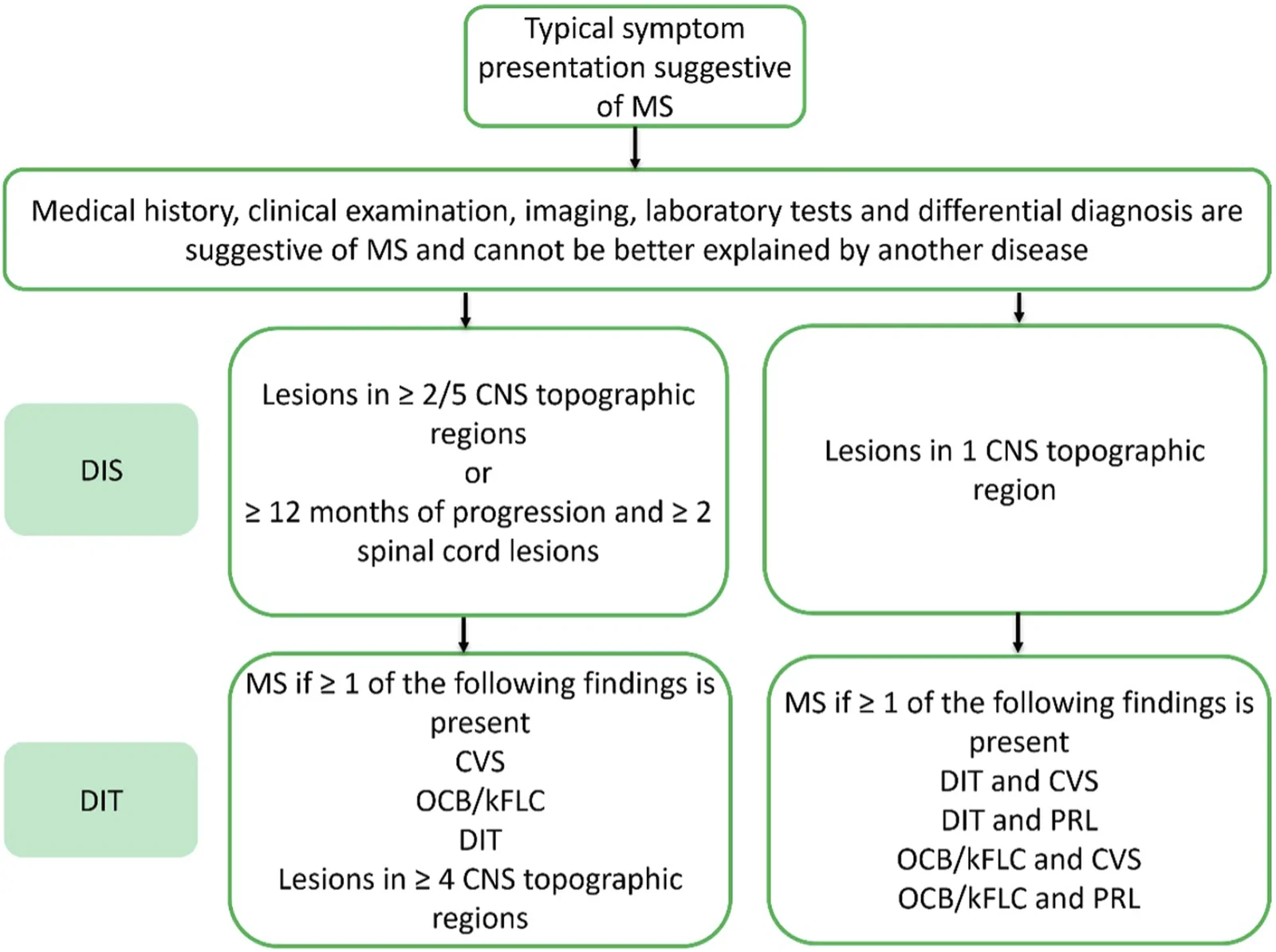
Diagnostic flowchart for MS based on the 2024 revised McDonald criteria.^5^ CNS: Central nervous system; CVS: Central vein sign; DIS: Dissemination in space; DIT: Dissemination in time; kFLC: Kappa free light chains; MS: Multiple sclerosis; OCB: Oligoclonal bands; PRL: Paramagnetic rim lesion.

The 2024 revision of the McDonald criteria introduces significant updates aimed at improving diagnostic accuracy and flexibility. In general, MRI has gained greater weight through the inclusion of additional MRI markers. Optic nerve lesions can be demonstrated by optic nerve MRI as fifth anatomical region for demonstration of dissemination in space. The detection of paramagnetic rim lesions (PRL) and lesions demonstrating the central vein sign (CVS) can serve as supportive features particularly in patients with lesions affecting one of five topographies suggestive of MS (Figure 1).^5^ Furthermore, patients presenting with incidental imaging findings suggestive of MS without any neurological symptoms suggestive of MS classified as a radiologically isolated syndrome (RIS) may be diagnosed with MS under specific conditions, reflecting a shift toward earlier recognition of disease biology prior to clinical manifestation.^5^ In CSF analysis, kappa free light chains (kFLC) are now recognized as an alternative biomarker to oligoclonal bands (OCB) for the detection of intrathecal humoral inflammation.^5^ The revised criteria also emphasize imaging and laboratory findings over purely clinical evidence, and in certain scenarios, DIT is no longer mandatory for diagnosis.^5^ Additional specifications address patients presenting after the age of 50 years and recommend myelin oligodendrocyte glycoprotein (MOG) antibody testing in pediatric cases.^5^ Finally, progressive and relapsing forms of MS are now unified under a single diagnostic framework, requiring harmonized criteria for all phenotypes.^5^

The consequent implementation of the 2024 McDonald criteria in the German healthcare system will require overcoming several practical challenges including misdiagnosis due to lack of knowledge and misapplication of criteria. The application of the new diagnostic criteria on regular basis in all eligible patients requires an expansion of the MR acquisition protocol including optic nerve imaging and optimized susceptibility-sensitive imaging. In addition, the image analysis, particularly regarding the detection of PRL and lesions with a CVS, requires additional time and dedicated expertise which is not broadly available. Established methods of optic nerve imaging such as visual evoked potentials and optical coherence tomography should also be included. Furthermore, the nationwide implementation of kFLC analysis is still evolving, as many institutions are continuing to build experience with this biomarker and not all laboratories currently offer testing in both serum and CSF.

This present point-of-view paper aims to discuss the practical implementation of the revised McDonald criteria in Germany and provide consensus recommendations from MS experts on a national level.

## Methods

### Panel composition

The consortium comprised nine multidisciplinary experts (eight board-certified neurologist and one board-certified neuroradiologist) specialized in the diagnosis and treatment of MS patients and/or MRI to discuss the implementation of the revised McDonald criteria in the clinical routine setting in Germany. All panel members were from eight institutions across Germany, six of which were affiliated with academic medical centers and two with private practice medical centers. The panel was founded and the panel members selected on the initiative of the chair (RG). The chair served as convenor and manuscript coordinator; topic leads were appointed for each predefined area including clinical presentation, imaging and blood as well as CSF biomarkers. Novartis Germany provided funding for the project, excluding any influence on topic selection, meeting conduct, or recommendation content by third parties.

### Meeting format and consensus process

On May 8^th^, 2025, a meeting was convened in Frankfurt am Main, Germany. The meeting was scheduled to allow pre-circulation of materials and to maximize participation. For each of the topic clinical presentation, imaging, electrophysiology and CSF biomarkers, a topic lead conducted a targeted literature review and prepared a 15-minute evidence summary slide presentation. Literature searches prioritized peer-reviewed studies, systematic reviews, and recent guideline statements relevant to the 2024 McDonald criteria and imaging biomarkers.

On each topic, recommendations were refined in real time during the meeting and subsequently circulated as a revised draft to all members for review. Discussions focused mainly on reproducibility, technical feasibility in routine clinical practice, and compatibility with the 2024 McDonald criteria. A modified Delphi-style approach without formal anonymity was applied to achieve consensus. A recommendation was considered accepted when a majority of the panel members agreed. Recommendations that did not meet the acceptance threshold were revised by the topic lead to address the identified concerns and the revised recommendations were subsequently recirculated.

### Integration and manuscript drafting

Outcomes from the topics discussed during the meetings were synthesized into a single set of recommendations. The manuscript was drafted by MPW supported by the topic leads. The manuscript was circulated for iterative comment and approved by all panel members before submission.

## Results and discussion

### Magnetic resonance imaging

MRI of the brain and spinal cord remains a cornerstone among paraclinical methods for MS diagnosis, prognostic classification and monitoring of MS.^6,7^ With the 2024 revision of the McDonald criteria, significant updates have been made to the MRI-based diagnostic framework. The presence of lesions in the optic nerve represents an additional location for the demonstration and dissemination in space. The presence of lesions with a CVS and PRLs is highly specific for MS and aid in differentiating MS from important differential diagnoses particularly lesions due to ischemic small vessel diease.^8–10^ Therefore the detection of these lesions facilitate the diagnosis of MS particularly in patients with typical symptoms and typical lesion(s) in only one single topography suggestive of MS and in asymptomatic patients with incidental imaging findings suggestive of MS. Along with structured guidance for atypical presentations and age-related changes, these updates aim to enhance both the sensitivity and specificity of diagnosis, particularly in patients with MRI lesions affecting one single topography suggestive of MS, atypical clinical presentations or in challenging contexts such as incidental imaging findings suggestive of MS classified as radiologically isolated syndrome (RIS), pediatric cases, or older adults with microvascular comorbidity.^11^ In addition, this includes patients with relevant comorbidities such as headache disorders, vascular risk factors, and other conditions that may complicate the differential diagnosis.

*Practical challenges:* The detection of lesions with CVS, PRLs and lesions affecting the optic nerve involvement demands access to dedicated neuroradiological expertise and an expansion of the MR acquisition protocols including dedicated imaging sequences such as optimized susceptibility weighted imaging (SWI) or T2* echo-planar imaging (T2* EPI) and optic nerve imaging. The expertise, particularly regarding the analysis of lesions with a CVS and PRLs is not broadly availably and limited to specialized centers.^12–14^ In addition, the subsequent increase in MR acquisition time and image analysis is difficult to implement in a clinical routine setting in the context of the German healthcare system, especially for outpatients. In addition, the additional time and effort regarding image acquisition, postprocessing and image analysis is not reimbursed by health care insurance.

**Figure 2.**
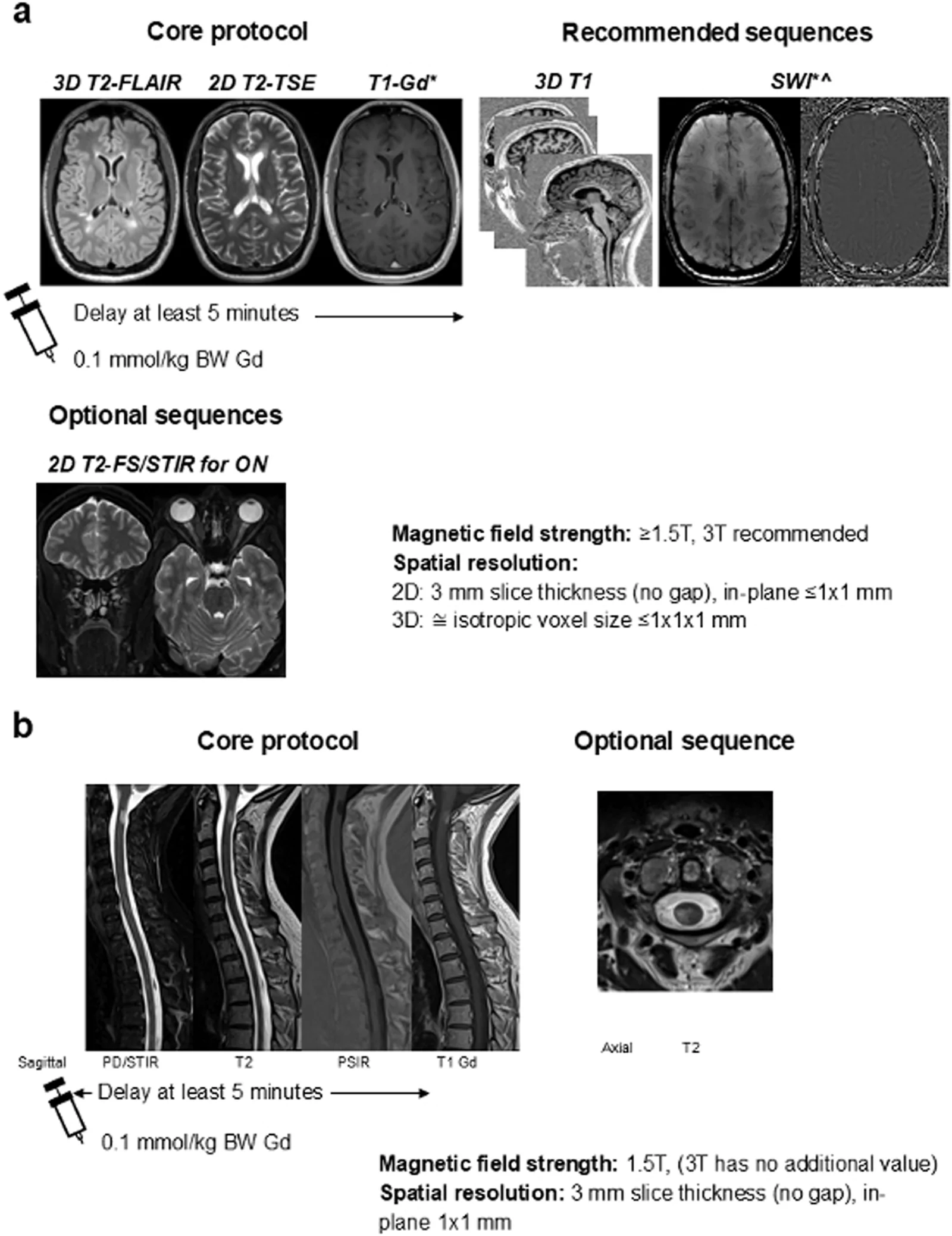
Core protocols, recommended and optional MRI sequences for use in neuroradiological practice according to the recommendation of the German Society of Neuroradiology.^18^ **a.** MRI protocols of the head for the diagnosis of MS. The 3D FLAIR can be acquired in sagittal or axial plane. Sagittal FLAIR allows the assessment of the perivascular distribution pattern of MS lesions. The susceptibility-sensitive imaging techniques can include T2*-EPI or optimized SWI acquisitions^18,19^ The 3D heavily T1-weighthed sequences such as MPRAGE or MP2RAGE allows the acquisition of volumetric data **b.** MRI protocols of the spinal cord for the diagnosis of MS. The use of PSIR is not routinely used. The replacement of STIR or PD-weighted imaged by PSIR in the clinical routine setting should be avoided. * 3D or 2D image acquisition ^ including phase image reconstruction. BW: Body Weight; FLAIR: Fluid Attenuated Inversion Recovery; FS: Fast Spin; Gd: Gadolinium; MRI: Magnetic Resonance Imaging; MS: Multiple Sclerosis; ON: Optic Nerve; PD: Proton Density; PSIR: Phase-sensitive Inversion Recovery; STIR: Short Tau Inversion Recovery; SWI: Susceptibility Weighted Imaging; T: Tesla; TSE: Turbo Spin Echo.

*Recommendations:* Considering these challenges, the risk of misdiagnosis of MS persists in clinical practice and may become even more relevant when less experienced centers will try to apply these new MRI markers for MS diagnosis. A significant factor contributing to this risk is the variability in knowledge and application of the McDonald criteria, particularly the putative focus on nonspecific MRI findings in patients with atypical symptoms.^15,16^ To address these challenges, the implementation of standardized MRI protocols according to the guidelines established by the German Society of Neuroradiology^17,18^ (Deutsche Gesellschaft für Neuroradiologie, DGNR) and endorsed by the German Society of Neurology (DGN) are crucial for the effective use of imaging biomarkers in routine clinical practice. In addition, it is important that these new MRI markers for MS diagnosis are used carefully and exclusively by centers that provide sufficient expertise in this field. For these purposes, the MRI acquisition protocol should consider core, recommended and optional pulse sequences (Figure 2). To balance diagnostic efficacy with efficient resource utilization a tiered diagnostic approach is recommended.

#### Optic nerve imaging

**Figure 3.**
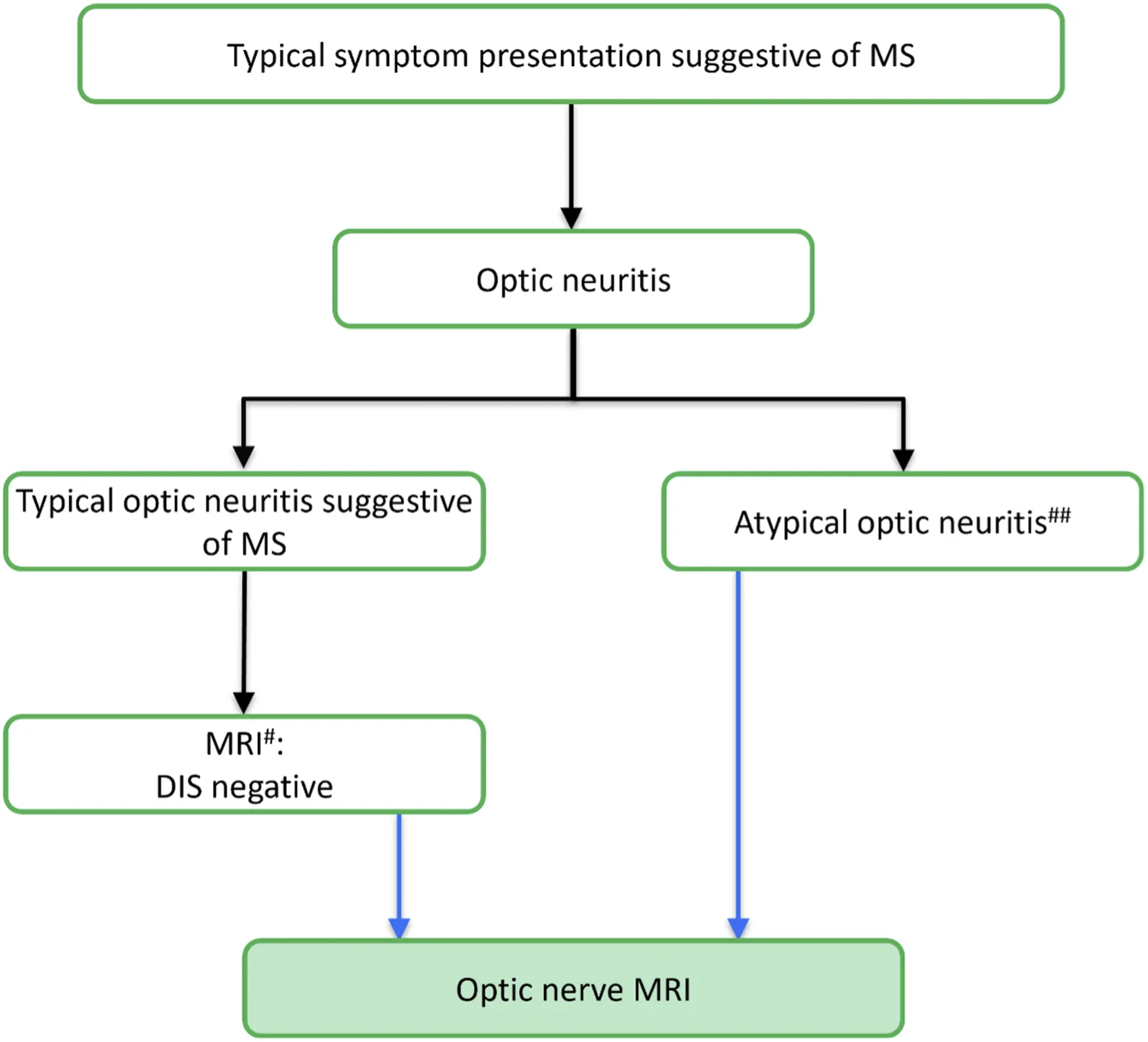
Flowchart on when optic nerve imaging is recommended to diagnose MS in patients with typical or atypical optic neuritis when OCT and VEP are not available according to the 2024 McDonald criteria and 2024 MAGNIMS-CMSC-NAIMS consensus recommendations.^5,19^^#^ compliant with 2024 MAGNIMS-NAIMS-CMSC consensus recommendations.^19^^##^ Typical optic neuritis refers to the clinical presentation classically associated with MS. Atypical presentations deviate from this profile and may be characterized by severe visual loss, severe or absent pain, lack of remission, or bilateral onset. Non-MS etiologies are further suggested by specific paraclinical features, including MRI findings (e.g., longitudinally extensive lesions, atypical lesion location or enhancement patterns), OCT metrics (e.g., degree of pRNFL swelling), or a dissociation between the extent of neurodegeneration and visual recovery. CMSC: Consortium of Multiple Sclerosis Centers; CNS: Central nervous system; DIS: Dissemination in space; MAGNIMS: Magnetic Resonance Imaging in Multiple Sclerosis; MRI: Magnetic Resonance Imaging; MS: Multiple Sclerosis; NAIMS: North American Imaging in Multiple Sclerosis; OCT: Optical Coherence Tomography; pRNFL: Peripapillary Retinal Nerve Fiber Layer.

Conducting an MRI of the optic nerve in patients with typical or atypical optic neuritis in the context of MS diagnosis related to the clinical presentation is recommended if OCT and visually evoked potentials (VEP) are not available or inconclusive, especially in unclear cases (Figure 3). Optic nerve involvement provides critical data regarding potential differential diagnoses, such as neuromyelitis optica spectrum disorders (NMOSD) or MOG antibody-associated disease (MOGAD).^19^ Please note that VEP may be more informative in the acute phase of optic neuritis, whereas OCT changes may not be evident in the first 6 months.

#### Novel MRI biomarker for the demonstration of lesions with a CVS and PRLs

**Figure 4.**
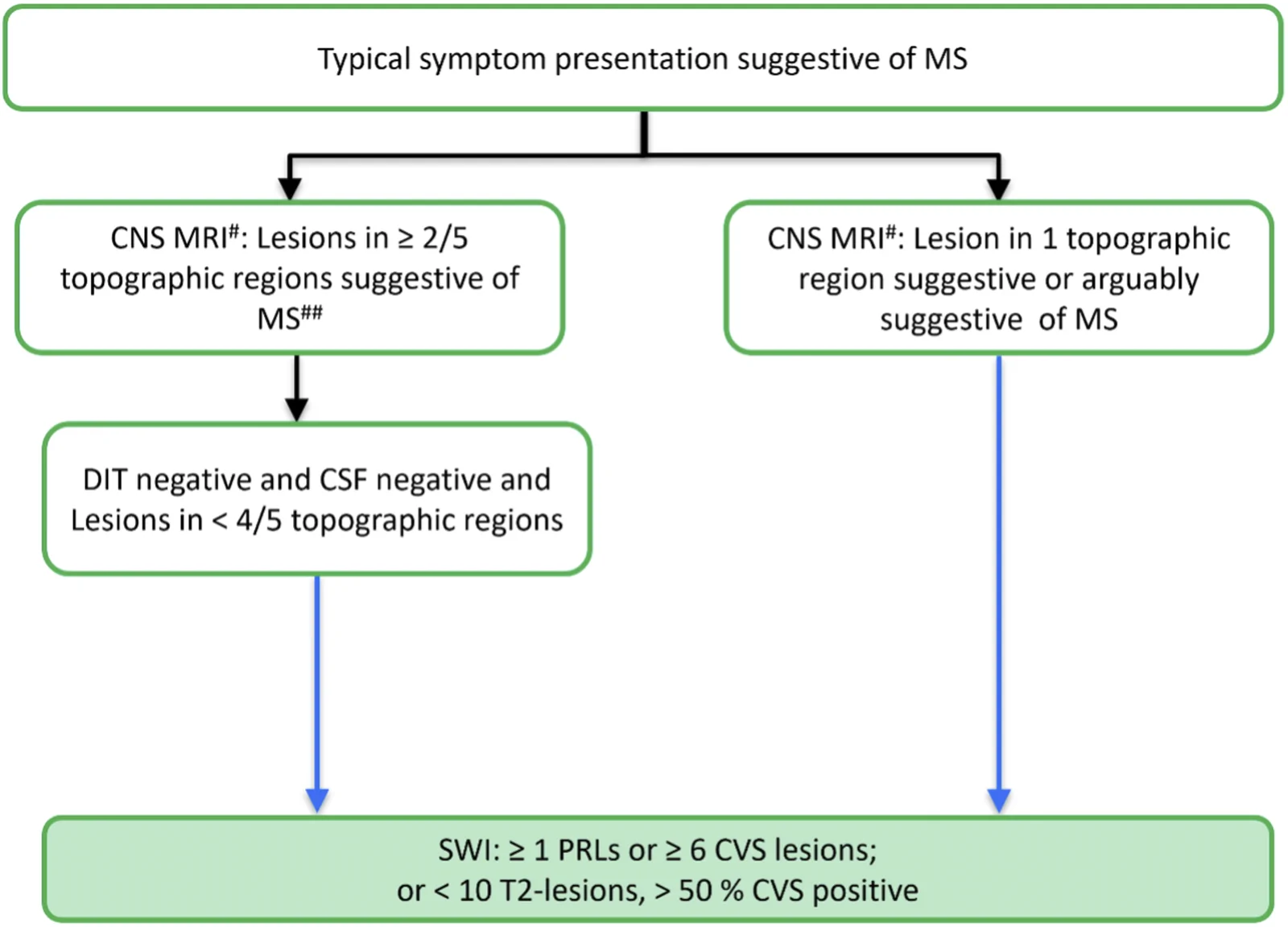
Flowchart illustrating the indications for SWI to detect CVS and/or PRLs in patients with relapsing or progressive clinical presentations, supporting the diagnosis of MS in accordance with the 2024 McDonald criteria and the 2024 MAGNIMS-CMSC-NAIMS consensus recommendations. Please not the special clinical relevance of these markers for patients older than 50 years or those with relevant comorbidities such as ischemic small vessel disease. ^#^ compliant with 2024 MAGNIMS-NAIMS-CMSC consensus recommendations.^19^^##^ according to the 2024 revision of the McDonald criteria.^5^ CMSC: Consortium of Multiple Sclerosis Centers; CNS: Central nervous system; CSF: Cerebrospinal fluid; CVS: Central vein sign; DIT: Dissemination in time; MAGNIMS: Magnetic Resonance Imaging in Multiple Sclerosis; MRI: Magnetic Resonance Imaging; MS: Multiple Sclerosis; NAIMS: North American Imaging in Multiple Sclerosis; PRL: Paramagnetic rim lesion; SWI: Susceptibility weighted imaging.

**Figure 5.**
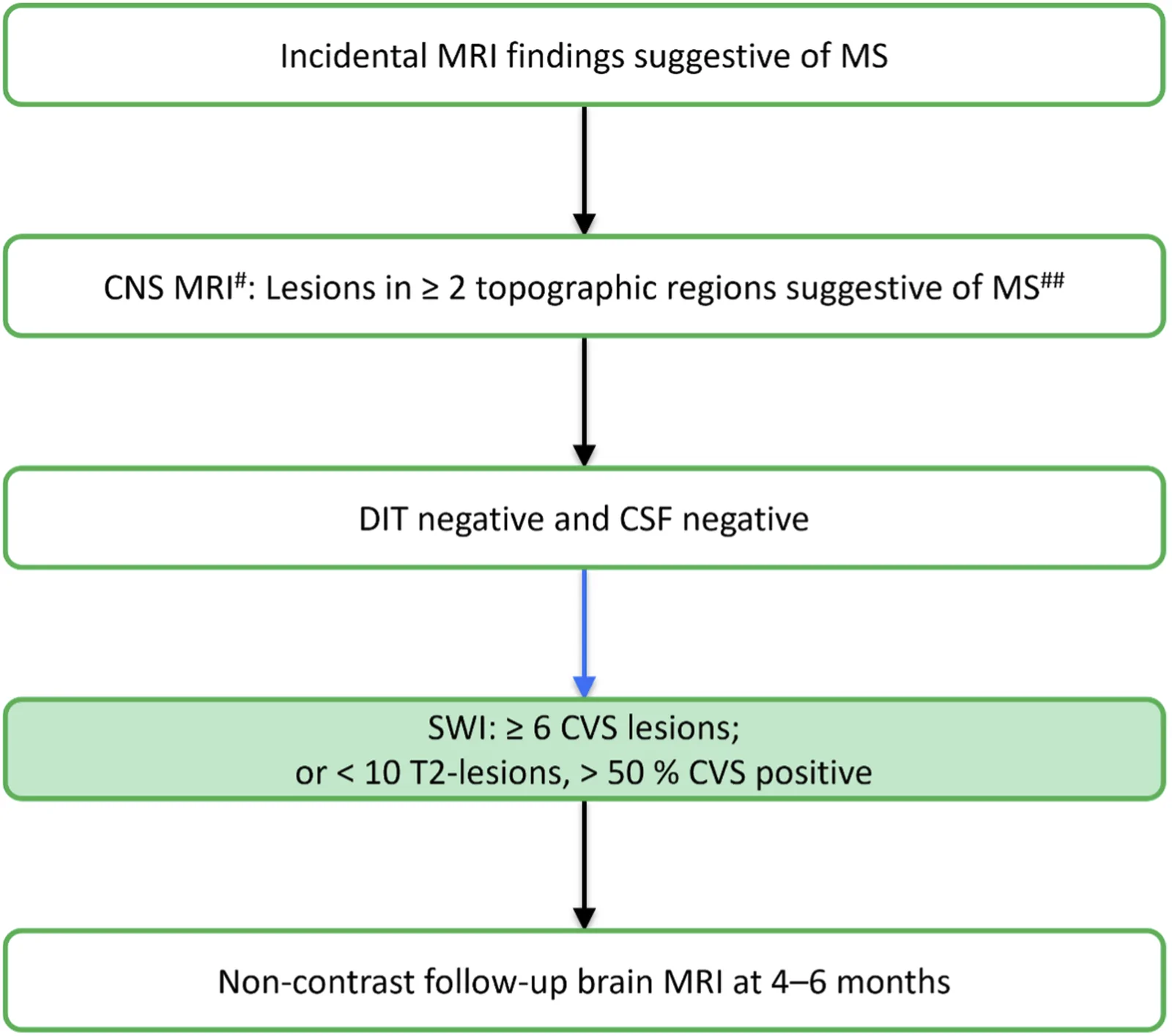
Flowchart illustrating the indications for SWI to detect CVS in patients with MRI presentations suggestive of MS in accordance with the 2024 McDonald criteria and the 2024 MAGNIMS-CMSC-NAIMS consensus recommendations. The flowchart further clarifies that a non-contrast follow-up brain MRI at 4–6 months is indicated for patients who are negative for both DIT and CSF, and who subsequently do not meet the specified CVS criteria on SWI. ^#^ compliant with 2024 MAGNIMS-NAIMS-CMSC consensus recommendations.^19^^##^ according to the 2024 revision of the McDonald criteria.^5^ CMSC: Consortium of Multiple Sclerosis Centers; CNS: Central nervous system; CSF: Cerebrospinal fluid; CVS: Central vein sign; DIT: Dissemination in time; MAGNIMS: Magnetic Resonance Imaging in Multiple Sclerosis; MRI: Magnetic Resonance Imaging; MS: Multiple Sclerosis; NAIMS: North American Imaging in Multiple Sclerosis; SWI: Susceptibility weighted imaging.

Routine inclusion of SWI in the standard assessment protocol is not recommended. Routine SWI can have relatively stronger T1-weighting, which may make T2-hyperintense lesions appear less conspicuous and is therefore insufficient for reliable CVS detection. T2*-EPI is more sensitive for detecting lesions with CVS and is now clinically available.^18^ In the case T2*-EPI is not available, the 2024 MRI recommendations also suggest the use of optimized SWI acquisitions with low flip angles.^19^ Instead, a stratified approach is proposed, reserving SWI for patients in whom identifying CVS and/or PRL alters the diagnostic outcome (Figures 4 and 5). However, at specialized MS centers, routine acquisition of susceptibility-sensitive imaging techniques may be considered to build local expertise and gain experience.

### Cerebrospinal fluid (CSF)-marker

The 2024 McDonald criteria consider kFLC in CSF as an alternative biomarker to OCB for demonstrating intrathecal humoral immune response.^5,20^ Similar to OCBs, the presence of kFLC is a characteristic feature of MS and can be used to differentiate MS from other mimics, particularly in settings where OCB testing is unavailable or inconclusive.^21^ kFLC analysis offers a rapid, automated and quantitative approach with reduced examiner dependency and lower labor intensity compared with OCB, while further standardization of interpretation and multicenter validation remain necessary.^22^ In addition, it remains to be determined whether kFLC is already positive at earlier stages of MS.

*Practical challenges:* To date, newer biomarkers such as kFLC lack fully standardized measurement procedures and established reference values. Variability in laboratory standards may result in false-negative and false-positive findings. As with OCB, kFLC results must always be interpreted within the appropriate clinical context, as intrathecal inflammation may also occur in conditions other than MS. The German Society for Cerebrospinal Fluid Diagnostics and Clinical Neurochemistry (DGLN e.V.) recommended a complementary approach using both CSF specific OCB and kFLC in paired CSF and serum samples. If only one method is available, OCB should be performed first, with additional kFLC testing in negative or borderline cases, or as an alternative when OCB testing is unavailable.^23^

*Recommendations:* CSF analysis should be performed in experienced laboratories, ideally combining OCB and kFLC.

### Costs and resources

*Practical challenges:* The implementation of additional imaging protocols and laboratory diagnostics results in a substantial increase in workload and associated costs, which are not reimbursed within the German health insurance framework. Furthermore, the availability of specialized diagnostic services such as expanded MRI protocols or optical coherence tomography (OCT) remains limited outside of specialized care centers across Germany.

*Recommendations:* Evidence-based, high-quality reporting remains essential even under resource constraints. Guidelines on the standardized application and interpretation of advanced imaging techniques and CSF biomarkers – while considering economic implications – can contribute to achieving this goal. Despite this, the 2024 McDonald criteria shift the focus toward a unified, “biological” definition of MS to facilitate earlier diagnosis. However, definitive validation studies in the clinical routine setting are currently limited and it can be anticipated that in the vast majority of patients presenting with suspected MS, the diagnosis of MS can be made with the approach implemented by the 2017 revisions of the McDonald criteria. Therefore, we consider the implementation of optic nerve (optic nerve MRI or OCT), PRLs and CVS not mandatory for diagnosis but provide valuable support in ambiguous cases and may potentially allow earlier diagnosis of MS.^5^ However, at specialized MS centers, routine acquisition of additional sequences may support the development of local expertise and facilitate the acquisition of practical experience.

### Education and expertise

*Practical challenges:* Another major concern of the McDonald criteria are the potential for misdiagnosis, particularly false positive MS diagnosis. Inaccurate diagnosis has been already a substantial issue with the use of the 2017 revisions of the McDonald criteria.^24^ Implementing the 2024 revisions of the McDonald criteria with additional imaging and fluid biomarkers and a quite complex diagnostic algorithm raises particularly due to the inclusion of RIS as a diagnostic entity the concern of a higher number of inaccurate diagnosis.^25^ In addition, there is still a lack of expertise and confidence using imaging features for MS diagnosis.^15,26^ To avoid these sources of potential error, structured and comprehensive training programs for neurologists and radiologists in the application of the new diagnostic criteria are needed.

*Recommendations:* In clinical practice, diagnostic uncertainty should prompt consultation with specialized centers for a second opinion. Continuous professional training for all involved disciplines – neurology, radiology, and, when applicable, laboratory medicine – is crucial to maintain diagnostic accuracy and reduce the risk of misdiagnosis. Thorough documentation of the applied criteria and the rationale behind clinical decisions is essential for transparency and quality assurance. In cases where the diagnosis remains unclear, close monitoring is preferable to premature initiation of treatment.

### Patient communication

*Practical challenges:* The 2024 revisions enable earlier diagnosis in individuals which were previously called RIS and atypical clinical presentations that previously fell outside the 2017 criteria. While this represents a major step forward, early diagnosis can create uncertainty for patients. As RIS diagnoses become more frequent, a corresponding rise in therapeutic interventions is anticipated; however, initiating treatment in asymptomatic individuals often proves difficult due to limited perceived benefit and poor adherence. Moreover, complex therapeutic decisions require thorough and individualized counseling to ensure informed choices.

*Recommendations*: In clinical practice, clear and transparent communication with patients about the benefits and limitations of the revised diagnostic criteria is essential. Special attention should be given to individuals with former (according to the 2017 McDonald^27^ and the 2009 or 2023 RIS^28,29^ criteria) RIS, ensuring that discussions address the potential stigma associated with MS and provide appropriate psychosocial support. This approach fosters trust, informed decision-making, and adherence to follow-up recommendations.

### Therapeutic consequences of the 2024 McDonald criteria

*Opportunities*: The 2024 revisions move beyond traditional concepts of DIS and DIT by introducing additional imaging and CSF biomarkers aiming to allow an earlier and more reliable diagnoses of MS. By providing more flexible pathways to diagnosis – including scenarios previously categorized as RIS – more individuals may be identified at prodromal and pre-symptomatic disease stages opening a window for earlier intervention with DMTs. In clinical practice, this also provides a clear therapeutic option without the risk of reimbursement penalties. Early initiation of treatment, particularly with high-efficacy therapies is associated with lower relapse rates, reduced disability progression, and higher rates of ‘no evidence of disease activity’ (NEDA) compared with delayed or escalation approaches, supporting a proactive therapeutic approach once diagnostic confidence is reached.^30–32^ Early use of highly effective therapies is especially recommended in patients with unfavorable prognostic factors, such as high lesion burden, early severe disability or positive biomarker profiles.^^7^ 33^

*Practical challenges:* With the 2024 McDonald criteria, the complexity of therapeutic decision-making is further increased. In addition, the new criteria place greater demands on patient communication and shared decision-making, especially in patients with atypical presentations or individuals without overt symptoms.

*Recommendations*: To avoid an inflationary approach to therapy, the indication for treatment must always be critically evaluated. The 2024 McDonald criteria should be applied in a nuanced manner, considering the overall clinical context rather than following a schematic interpretation. Therapeutic decisions should be individualized and made in accord with the patient, considering prognostic factors, e.g., spinal and Gd+ lesions, lifestyle circumstances, and personal preferences to ensure both clinical appropriateness and patient-centered care.

## Conclusion

The expert panel reached a consensus on the practical implementation of the 2024 McDonald criteria, emphasizing several key points.

A central conclusion is the affirmed role of CSF analysis, which remains essential for a reliable MS diagnosis in Germany. Important differential diagnoses mimicking MS might show simultaneous presence of Gd enhancing and non-enhancing lesions (e.g., cerebral lymphoma, vasculitis). Consequently, a diagnosis without CSF confirmation should remain an exception and is viewed critically.

While new MRI techniques such as optic nerve lesions, PRLs, and lesion showing a CVS as well as new CSF biomarkers such as kFLC are valuable additions, their interpretation requires specialized expertise. The panel stressed that the revised criteria must not lead to an inflation of diagnoses; clinical plausibility and high-quality findings are paramount. To ensure accurate application and prevent misinterpretation, continuous education and structured training are necessary. In cases of uncertainty, a second opinion from an experienced center is strongly recommended.

Ultimately, while the new 2024 revisions of the McDonald criteria may facilitate earlier therapeutic intervention, they also introduce greater complexity to clinical decision-making. This presents a multidimensional challenge for the German healthcare system, likely increasing diagnostic demands.
